# Supplementary material for: Polyketide synthase gene clusters in the ericoid mycorrhizal fungus Oidiodendron maius: genome-wide prediction, expression profiling, and functional characterization of knockout mutants
Source: Front Plant Sci. 2026 Sep 16;17:1924430. doi: 10.3389/fpls.2026.1924430 (PMC13623903; doi:10.3389/fpls.2026.1924430)
Supplement: Supplementary file 1 [file SupplementaryFile1.zip › Supplementary Materials/Supplementary Folder 1.zip!/Oidma1_AssemblyScaffolds_Repeatmasked/knownclusterblast/cluster16/jgi.p_Oidma1_195844_mibig_hits.html]

| MIBiG Protein | Description | MIBiG Cluster | MiBiG Product | % ID | % Coverage | BLAST Score | E-value |
| --- | --- | --- | --- | --- | --- | --- | --- |
| AGO59052.1 | PtaK | BGC0000121 | Pestheic\_acid\_biosynthetic\_gene\_cluster | 43 | 80.0963081862 | 416 | 4.1e-116 |
| EFY94489.1 | laccase\_Lcc4 | BGC0000337 | Destruxins\_biosynthetic\_gene\_cluster | 34 | 92.6163723917 | 309 | 7e-84 |
| AGO59042.1 | PtaE | BGC0000121 | Pestheic\_acid\_biosynthetic\_gene\_cluster | 31 | 85.2327447833 | 257 | 4.1e-68 |
| EAL89346.1 | extracellular\_dihydrogeodin\_oxidase/laccase,\_putative | BGC0001403 | Trypacidin\_biosynthetic\_gene\_cluster | 36 | 59.5505617978 | 243 | 8.1e-64 |
| ADB23378.1 | laccase | BGC0001062 | TLN-05220\_biosynthetic\_gene\_cluster | 39 | 23.7560192616 | 99 | 1.9e-20 |
| EWM63046.1 | multicopper\_oxidase | BGC0000679 | Diazepinomicin\_biosynthetic\_gene\_cluster | 35 | 26.9662921348 | 90 | 6.7e-18 |
